# Idiopathic systemic capillary leak syndrome: a case report

**DOI:** 10.1186/s12882-023-03122-4

**Published:** 2023-03-25

**Authors:** Hyang-yun Lee, Jungho Shin, Su-Hyun Kim, Jin Ho Hwang

**Affiliations:** 1 Division of Nephrology, Department of Internal Medicine, Chung-Ang University Hospital, Seoul, Korea; 2 Department of Internal Medicine, Chung-Ang University College of Medicine, Chung-Ang University Hospital, 102, Heukseok-Ro, Dongjak-Gu, Seoul, 06973 Republic of Korea; 3 Division of Nephrology, Department of Internal Medicine, Chung-Ang University Gwangmyeong Hospital, Gyeonggi-Do, Korea

**Keywords:** Acute kidney injury, Case report, Hemoconcentration, Hypoalbuminemia, Idiopathic, Systemic capillary leak syndrome

## Abstract

**Background:**

Idiopathic systemic capillary leak syndrome (ISCLS) is a rare disease characterized by recurrent episodes of acute life-threatening attacks of shock, hemoconcentration, and hypoalbuminemia. Increase in capillary permeability results in reversible plasma movement into the interstitial spaces followed by appearance of related symptoms or complications, including renal failure. This condition can be potentially life-threatening; however, it is easily misdiagnosed.

**Case presentation:**

A 47-year-old man with no previous medical history presented to the emergency department after experiencing general weakness and abdominal pain. He developed hypovolemic shock within 3 h of presentation and initial laboratory tests showed hemoconcentration, hypoalbuminemia and acute kidney injury. Following vigorous fluid therapy and supportive care, the patient recovered, but a similar episode recurred after 4 months without any specific trigger. Based on the combined clinical manifestations and laboratory findings of both the attacks, he was diagnosed with ISCLS. Symptomatic relief was achieved via oxygen supplementation and massive volume replacement using normal saline and the patient was prescribed bambuterol 10 mg and theophylline 400 mg once-a-day. He was discharged from the hospital on day 5 of hospitalization. Thereafter, the patient has been followed for 5 years without any symptoms or recurrence of ISCLS even in the situation of COVID-19 infection.

**Conclusions:**

ISCLS is an extremely infrequent and commonly misdiagnosed disease. However, early diagnosis, treatment and prophylaxis through accumulated clinical data can prevent ISCLS recurrence and the development of related fatal complications. Therefore, clinicians need to be well aware of the variety of clinical characteristics and treatment options of this disease.

## Background

Idiopathic systemic capillary leak syndrome (ISCLS) is a rare and fatal disease, first described by Clarkson in 1960. The first case of ISCLS was reported in a 32-year-old woman who experienced periodic sudden and unexplained movement of plasma from intravascular compartment to interstitial space due to increased capillary permeability [1]. ISCLS is characterized by recurrent episodes of edema, hypovolemic shock, hypoalbuminemia, and hemoconcentration. Monoclonal gammopathy is common among patients with SCLS, which guides the diagnosis but is not considered a major diagnostic criterion [2]. Other possible differential diagnoses include polycythemia vera, sepsis, anaphylaxis, and reactions to certain drugs and toxins [2, 3].

Since ISCLS is rarely encountered in clinical practice and shows a variety of manifestations, it is difficult to differentiate from other disorders, which might cause a delay in diagnosis and development of life-threatening complications. In patients with ISCLS, acute kidney injury (AKI) is a common complication that sometimes culminates to chronic renal failure requiring hemodialysis [3–7]. Herein, we report a case of ISCLS with recurrent AKI that was successfully controlled with theophylline and long-acting oral β2-agonist administration.

## Case presentation

### Clinical history and initial laboratory data

A 47-year-old man was admitted to our hospital after presenting to the emergency department in March 2017, with complaints of general weakness, lower back pain and abdominal pain that started on the same day. The patient had no underlying diseases or history of taking any medications. He was a never-smoker, and social drinker. There were no significant recent changes in his lifestyle or diet, except overworking.

The patient was alert and only a dry tongue and axilla were observed during physical examination. His vital signs were stable at presentation, but the blood pressure decreased to 74/60 mmHg, 3 h after arrival in the emergency room. Initial laboratory tests revealed several abnormalities (Table 1), including an elevated white blood cell count (WBC) of 29,180 × 10^6^ cells/L, hemoglobin (Hgb) of 22.6 g/dL, hematocrit (Hct) of 66.1%, blood urea nitrogen (BUN) of 28 mg/dL, creatinine (Cr) of 1.95 mg/dL, fractional excretion of sodium (FENa) of 0.13%, hypoalbuminemia with serum albumin level of 2.6 g/dL, C-reactive protein (CRP) level of 7.7 mg/L, and procalcitonin level of 0.8 ng/ml. Cardiac markers were normal (creatine kinase-MB = 0.94 ng/ml, troponin-I < 0.006 ng/ml). Arterial blood gas analysis (ABGA) showed normal anion gap metabolic acidosis with respiratory compensation (pH = 7.352, PaCO_2_ = 24.5 mmHg, PaO_2_ = 117 mmHg, HCO_3_ = 13.3 mmHg, and lactic acid = 3.0 mmol/L). Non-enhanced abdominal and pelvic computed tomography (CT) scans showed normal sized kidney and borderline splenomegaly. Doppler echocardiography showed normal global left ventricular (LV) systolic function, concentric LV hypertrophy, and minimal amount of pericardial effusion. Owing to the elicitation of polycythemia on blood examination, bone marrow biopsy was performed based on the suspicion of a reactive marrow, which revealed no abnormalities or JAK2 gene V617F mutation. Serum protein electrophoresis returned normal results and antinuclear antibody was negative.

**Table 1:** Laboratory data on admission

Parameter	Unit	1st admission	2nd admission	Reference range
White blood cell [WBC]	10^9^/L	29.2	30.2	3.0—9.0
Hemoglobin [Hgb]	g/dL	22.6	19.7	13—17
Hematocrit [Hct]	%	66.1	59.3	40—50
blood urea nitrogen [BUN]	mg/dL	28	39	8—9
Creatinine [Cr]	mg/dL	1.95	2.93	0.67—1.17
Sodium [Na]	mEq/L	134	126	135—146
Potassium [K]	mEq/L	4.8	6.6	3.5—5.3
Albumin [Alb]	g/dL	2.6	3.6	3.3—4.9
C-reactive protein [CRP]	mg/L	7.7	1.1	0—5.0
procalcitonin	ng/mL	0.8	N/C	0—0.5
CK-MB	ng/mL	0.94	0.91	0—5
Troponin-I	ng/mL	< 0.006	< 0.006	0—0.78
JAK2 gene V617F mutation		Negative		-
ANA-titer		Negative		-
serum protein electrophoresis		Normal		-
serum Free light chain—Kappa	mg/L		32.3	3.3—19.40
serum Free light chain—Lambda	mg/L		15.7	5.71—26.30
serum Free light chain—K/L ratio			2.057	-
IgG	mg/dL		1,224	-
- IgG1	mg/dL		909	-
- IgG2	mg/dL		206	-
- IgG3	mg/dL		36.5	-
- IgG4	mg/dL		0.4	-
IgA	mg/dL		150	-
IgM	mg/dL		64	-

### Clinical course

Features of dehydration coupled with the laboratory findings suggested pre-renal AKI and hypovolemic shock caused by decreased effective circulating volume. Despite administration of massive volume resuscitation and diuretics, severe hyperkalemia (serum potassium = 7.8 mEq/L), anuria and recurrent hypotension ensued. Ceftriaxone was started as an empirical antibiotic as the cause of shock was uncertain and WBC count as well as CRP levels were elevated. On the second day of hospitalization, the patient complained of pain, redness and swelling in the left lower leg. Thereafter, an ultrasonography was performed, which ruled out deep vein thrombosis; however, subcutaneous edema was evident. As these findings suggested cellulitis, ceftriaxone was switched to cefazolin. The patient recovered dramatically on the third day of hospitalization and was discharged on the tenth day with WBC count of 9,080 × 10^6^ cells/L, Hgb of 13.4 g/dL, BUN/Cr of 9/0.81 mg/dL and albumin of 4.2 g/dL. He remained symptomless for 3 months after discharge.

Three months after the last admission, the patient visited our hospital again because of sudden dizziness and dyspnea. On admission, the blood pressure was 86/50 mmHg, pulse rate was 104/min, respiratory rate was 20/min, and his body temperature was 36.8℃. The laboratory results showed an increased WBC of 30,150 × 10^6^ cells/L, Hgb of 19.7 g/dL, Hct of 59.3%, BUN/Cr of 39/2.93 mg/dL and decreased albumin level of 3.6 g/dL. The levels of CRP (1.1 mg/L) and cardiac markers (creatine kinase-MB = 0.91 ng/ml, Troponin-I < 0.006 ng/ml) were within normal limits. The FENa was 0.1% suggesting pre-renal AKI. The ABGA showed normal anion gap metabolic acidosis with respiratory compensation (pH = 7.34, PaCO_2_ = 24.6 mmHg, PaO_2_ = 142 mmHg, HCO_3_ = 12.9 mmHg, and lactic acid = 2.4 mmol/L). Kappa and lambda free light chains were 32.3 and 15.7 mg/dL, respectively, and the Kappa/lambda ratio was 2.057. Total serum IgG was 1224 mg/dL, and subclass distribution was a s follows: IgG1 of 909.0, IgG2 of 206.0, IgG3 of 36.5, and IgG4 of 0.4 mg/dL. Serum protein electrophoresis results was normal and non-enhanced chest CT showed no abnormalities.

**Fig. 1:**
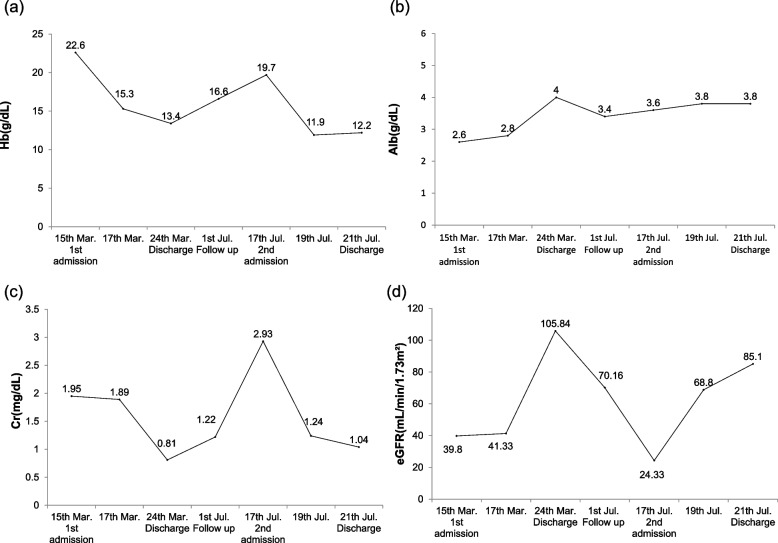
**a** Hemoglobin, **b** albumin, **c** creatinine, and **d** eGFR values during first and second attacks

**Fig. 2:**
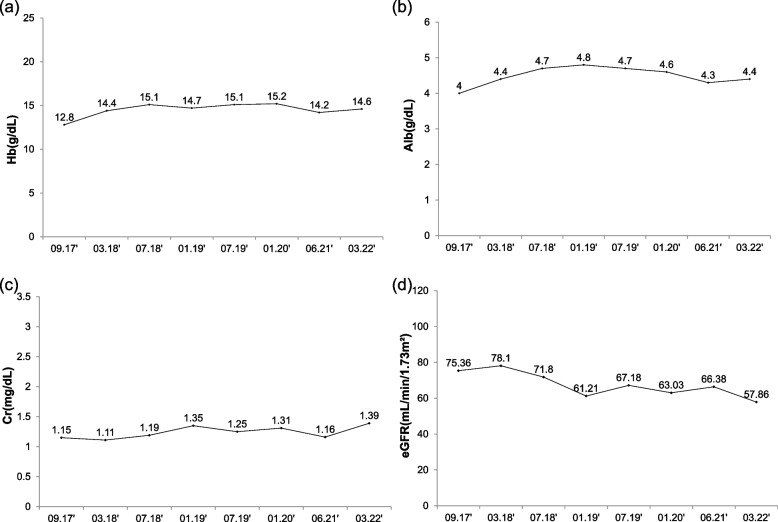
**a** Hemoglobin, **b** albumin, **c** creatinine, and **d** eGFR values during follow up

A clinical diagnosis of ISCLS was made on the basis of recurring clinical presentation of hypotension, hemoconcentration, and hypoalbuminemia, after excluding other reasons of shock, such as septic, cardiogenic, bleeding and toxin. His symptoms improved greatly after oxygen supplementation and massive volume replacement with normal saline. The hemoconcentration and AKI also resolved within the next few days (Fig. 1, a-d). Based on the previous studies, bambuterol 10 mg (long-acting oral β2-agonist) and theophylline 400 mg once-a-day were prescribed on day 3 of hospitalization [4, 5]. He was discharged from the hospital on day 5 post-admission, after being instructed to continue the same doses of oral bambuterol and theophylline. After 4 months of discharge, he experienced headache and flank discomfort suggesting a prodrome of acute attack of ISCLS. However, laboratory investigations, did not reveal significant abnormalities. The bambuterol dose was increased from 10 to 15 mg and the patient has been followed for 5 years without any symptoms or recurrence of ISCLS. As COVID-19 vaccination is not recommended for SCLC patients, he was followed by strict precaution without vaccination. He experienced COVID-19 infection in June 2022, but recovered well without SCLS episode while continuing the same medication he had been maintaining. The patient’s Hgb, albumin, Cr, and estimated glomerular filtration rate (eGFR) values during the follow up are shown in the Fig. 2a-d.

## Discussion and conclusion

ISCLS is a very rare disorder characterized by recurrent episodes of shock, hypoalbuminemia, and hemoconcentration. It is caused by a dysfunction of the vascular endothelium that leads to shift of fluid and proteins from the intravascular space to the interstitial space [1–3, 8].

The patient in the present case experienced two similar fatal attacks of hypovolemic shock, AKI, hypoalbuminemia, and hemoconcentration within a short duration of 4 months. The initial laboratory results showed normal serum protein electrophoresis and there was no other evidence of hematologic disorder on bone marrow biopsy. Although cellulitis was diagnosed, it was not considered as a precipitant factor of the first attack because it was mild and developed a few days after onset of the attack. Moreover, during the second attack, the final diagnosis of ISCLS was made after excluding all other possible causes of shock.

At present, there is no known curative treatment for ISCLS [3, 4, 6, 9]. The capillary leak phase with hypovolemia and hypotension is managed by vigorous fluid resuscitation, while in the recovery phase, diuretics are used to improve generalized edema and oliguria, and hemodialysis may be considered in severe cases [10]. In addition to conservative treatment, some prophylactic treatments have also been attempted. The pathophysiology of ISCLS is uncertain but it has been shown that macromolecular leakage in response to various stimuli, including histamine and bradykinin, can be inhibited by pretreatment with beta-2 stimulants like terbutaline-selective beta-2 stimulant and isoprenaline [4]. Combination of terbutaline and theophylline have shown good prognosis in some case series [4, 5, 8]. In addition, several studies have demonstrated better efficacy of intravenous immunoglobulin therapy (IVIG) than β2-agonists. Thus, IVIG has emerged as the most commonly used first-line prophylactic therapy in ISCLS and is effective at a dose of 2 g/kg once a month. [5, 6, 9]. However, in the present case, we could not prescribe IVIG due to high cost of treatment (29 USD for 1 g) and limitation of medical reimbursement system in Korea. The patient in this case reported no recurrence after initiating combination treatment with theophylline and bambuterol. However, if the patient experiences another attack in the future, IVIG infusion should be considered.

Previous case reports have described a range of complications associated with ICSLS [2, 7, 11]. One such report documenting a case of severe chronic ISCLS requiring daily hemodialysis, stated AKI as one of the most common manifestations of capillary leak syndrome [7]. Another study suggested intravascular volume depletion and cytokine-induced injury as contributors of acute tubular necrosis [2]. In our case, the patient experienced AKI in every attack of ISCLS. Although renal function fully recovered after the first attack, the eGFR remained below 70 ml/min/m^2^ after the second attack as shown in Fig. 2. This suggested that recurrent ISCLS attacks cause gradual deterioration of renal function, eventually resulting in part-permanent impairment.

**Fig. 3:**
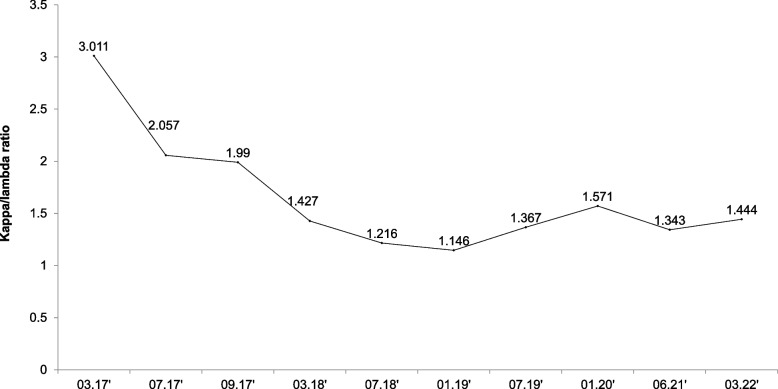
The trend of Kappa/Lambda ratio

Several studies have found monoclonal gammopathy of undetermined significance and multiple myeloma to be associated with SCLS [8]. In the present case, M-protein was not detected in the serum, but kappa/lambda was too high to be attributed to AKI alone. The patient received regular monitoring for multiple myeloma based on the recommendation of a previous case report that showed progression to multiple myeloma after diagnosis of the systemic capillary leak syndrome [6, 9]. In our case, serum kappa/lambda ratio had normalized over time and remained within normal range as shown in Fig. 3.

**Fig. 4:**
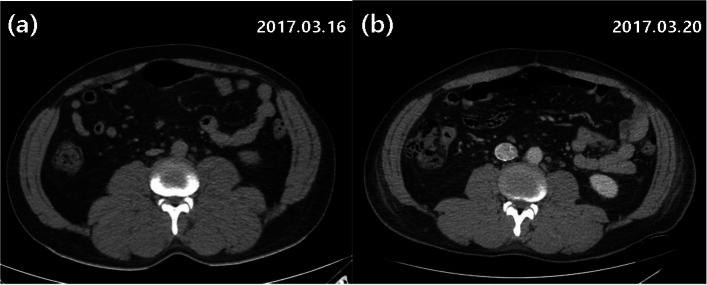
**a** Abdomen and pelvis CT demonstrates generalized edema of abdominal wall muscles. **b** Decreased muscle edema on subsequent chest CT 4 days later

In the present case, the patient complained of abdominal and lower back pain, but pain focus was unclear on the initial abdominal and pelvic CT examination (Fig. 4a). The only notable feature was thickening of abdominal wall and muscles, especially psoas major and erector spinae. However, the pain resolved spontaneously within a few days and follow-up CT after 4 days showed intermittent improvement in muscle edema (Fig. 4b). These findings suggested that muscle edema by capillary leak could trigger pain and even develop into compartment syndrome Also, compartment syndrome by capillary leak has been described in a few case reports in the past [11–14].

During the COVID-19 pandemic, a few cases were reported where patients experienced an episode of systemic capillary leak syndrome following COVID-19 vaccination or infection, especially those with a history of SCLS [15, 16]. According to European Medicines Agency, COVID-19 vaccination with adenoviral vectors is not recommended for patients with a history of SCLS. Since COVID-19 vaccination or infection can act as triggers for SCLS recurrence [17], he was followed by strict precaution without vaccination. He contracted COVID-19 infection in June 2022, but recovered well without SCLS episode while continuing the same medication he was maintaining. The relatively simple, inexpensive, and easy-to-adapt maintenance treatment he took once a day effectively prevented the recurrence of ISCLS episodes in usual times, and also effectively prevented the occurrence of SCLS episodes even when the risk was increased due to acute viral infection.

In conclusion, we reported a case of a patient with ISCLS who experienced two severe episodes of the disease, and has been followed for 5 years without recurrence even in the situation of COVID-19 infection, after being prescribed bambuterol and theophylline. ISCLS is a very rare pathology to come across for a clinician and it takes a long time to be diagnosed because other possible causes need to be excluded before reaching a diagnosis. Early diagnosis, treatment and prophylaxis through accumulated clinical data can prevent ISCLS from recurring as well as the development of various associated complications. Therefore, clinicians should be aware of the different clinical characteristics and treatment options of ISCLS.

## Data Availability

Records and data pertaining to this case are in the patient’s secure medical records in the Chung-Ang University Hospital. If needed, the relevant material can be provided by corresponding author on reasonable request.

## Abbreviations

ISCLS: Idiopathic systemic capillary leak syndrome
LV: Left ventricular
WBC: White blood cell
Hgb: Hemoglobin
Hct: Hematocrit
BUN: Blood urea nitrogen
Cr: Creatinine
FeNa: Fractional excretion of sodium
CRP: C-reactive protein
ABGA: Arterial blood gas analysis
CT: Computed tomography
IgG: Immunoglobulin G
AKI: Acute kidney injury
IVIG: Intravenous immunoglobulin therapy
